# Cardiovascular Effect Is Independent of Hemolytic Toxicity of Tentacle-Only Extract from the Jellyfish *Cyanea capillata*

**DOI:** 10.1371/journal.pone.0043096

**Published:** 2012-08-15

**Authors:** Xiao Liang, Wang Beilei, Li Ying, Wang Qianqian, Liu Sihua, Wang Yang, Liu Guoyan, Lu Jia, Ye Xuting, Zhang Liming

**Affiliations:** 1 Department of Chemical Defense Medicine, Faculty of Naval Medicine, Second Military Medical University, Shanghai, China; 2 School of Nursing, Second Military Medical University, Shanghai, China; 3 Department of Pathology, Changhai Hospital, Second Military Medical University, Shanghai, China; 4 Department of Biophysics, School of Basic Medical Sciences, Second Military Medical University, Shanghai, China; Cliniche Humanitas Gavazzeni, Italy

## Abstract

Our previous studies have confirmed that the crude tentacle-only extract (cTOE) from the jellyfish *Cyanea capillata* (Cyaneidae) exhibits hemolytic and cardiovascular toxicities simultaneously. So, it is quite difficult to discern the underlying active component responsible for heart injury caused by cTOE. The inactivation of the hemolytic toxicity from cTOE accompanied with a removal of plenty of precipitates would facilitate the separation of cardiovascular component and the investigation of its cardiovascular injury mechanism. In our research, after the treatment of one-step alkaline denaturation followed by twice dialysis, the protein concentration of the treated tentacle-only extract (tTOE) was about 1/3 of cTOE, and SDS-PAGE showed smaller numbers and lower density of protein bands in tTOE. The hemolytic toxicity of tTOE was completely lost while its cardiovascular toxicity was well retained. The observations of cardiac function, histopathology and ultrastructural pathology all support tTOE with significant cardiovascular toxicity. Blood gas indexes and electrolytes changed far less by tTOE than those by cTOE, though still with significant difference from normal. In summary, the cardiovascular toxicity of cTOE can exist independently of the hemolytic toxicity and tTOE can be employed as a better venom sample for further purification and mechanism research on the jellyfish cardiovascular toxic proteins.

## Introduction

Jellyfish stings can produce a burning feeling, severe pain, swelling, red streak, nausea, abdominal pains, profuse sweating, muscle cramp, respiratory distress, heart failure and so on [1]. Correspondingly, jellyfish venoms have a wide spectrum of biological activities, such as dermonecrotic, neurotoxic, hemolytic and cardiovascular activities [1], [2], [3]. It is believed that the effects of jellyfish venoms are caused by the combination of various toxic components [2], [3], and acute heart failure is recognized as the major cause of death caused by jellyfish venoms [4], [5], [6], [7], [8], where the cardiovascular toxic component may be the major damage factor with other toxic components, for example the hemolytic toxic component, acting in synergy with it [9], [10], [11].

Cardiovascular toxicity, the major toxic index of jellyfish venoms, is considered to be much more sensitive and selective to the cardiovascular system than to other physiological systems. It can be determined by an easy hypotensive response which is mainly related to heart failure [6], [7], [8], though the contractive vascular effect might also be influenced [12], [13]. Researchers have paid much more attention to the investigation on purification and identification of the cardiovascular toxic component [14], [15], action mechanism of the cardiovascular effect [16], [17], and prevention and therapy for the heart injuries by the cardiovascular toxicity [18], [19], [20]. Unfortunately, none of the above issues has now been settled satisfactorily.

Hemolysin, another greatly concerned jellyfish toxin, is present in different kinds of jellyfish venoms, and also is the only kind of jellyfish toxin which has been purified and identified successfully from the jellyfish *Carybdea rastoni* [21], *Carybdea alata* [22], *Chiropsalmus quadrigatus* [23], and *Chironex fleckeri* [24]. According to the sequence bioinformatic analysis and the investigations of hemolytic drug intervention [25], [26], hemolysin may function as a pore-former on cell membrane both *in vivo* and *in vitro* [27], [28], [29], [30]. Thus, hemolysin may contribute to the cardiovascular effect of jellyfish venoms through two pathways: the direct cytotoxicity [28], [29], [30] or the indirect damage on the heart by elevating K^+^ and lactic acid as a result of a large number of erythrocytes lysed [10], [31].

As a result, it would be quite difficult to determine the proportions of the cardiovascular toxic component and other toxic components, for instance hemolysin, in the cardiovascular effect of jellyfish venoms if crude venoms, with both cardiovascular toxicity and hemolytic toxicity, were used as the sample. Moreover, it is necessary to further confirm where the specific cardiovascular toxic component is located, and also whether the cardiovascular toxicity is independent of the hemolytic toxicity in jellyfish venoms. If hemolytic toxicity can be inactivated by an easy method accompanied with a removal of plenty of precipitates from jellyfish venoms with their cardiovascular toxicity retained, it will facilitate the purification of the cardiovascular toxic component, as well as improve the mechanism research on the cardiovascular toxicity.

In the present study, the hemolytic toxicity of the crude tentacle-only extract (cTOE) from the jellyfish *Cyanea capillata* (Cyaneidae), identified by Professor Huixin Hong from the Fisheries College of Jimei University, Xiamen, China, was inactivated successfully with a removal of plenty of precipitates just by one-step alkali denaturation followed by twice dialysis against two different buffer solutions, according to our previous experimental results [32], [33]. The observations of cardiac function both *in vivo* and *in vitro*, histopathology, hematology and ultrastructural pathology by transmission electron microscopy all support the cardiovascular toxicity of the treated extract (tTOE). It is concluded that the cardiovascular effect of tTOE is independent of the hemolytic toxicity of cTOE from the jellyfish *C. capillata*, and the tTOE can be used as the better venom sample for further purification and mechanism research.

## Results

### Determination of the Cardiovascular and Hemolytic Toxicities

**Figure 1:**
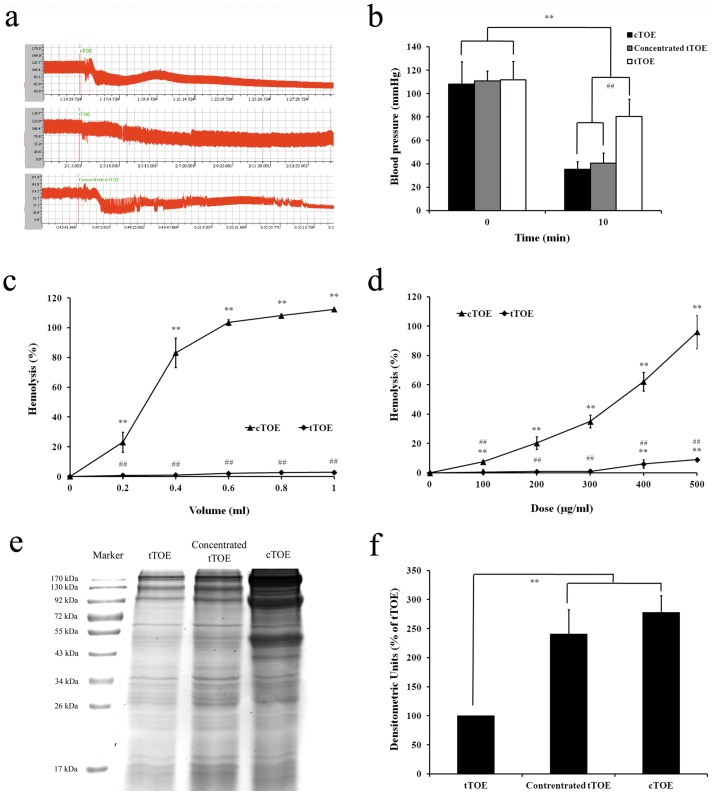
Determination of the cardiovascular and hemolytic toxicities of cTOE and tTOE from the jellyfish *C. capillata*. (a) Effects of cTOE (10 mg/kg), tTOE (3.3 mg/kg) and concentrated tTOE (10 mg/kg) on the blood pressure of SD rats (n = 6); (b) Comparison of the changes of blood pressure at 10 min after administration of cTOE and tTOE (n = 6; ^**^P<0.01 means the blood pressure before injection *vs.* that at 10 min after injection in the same group; *^##^P*<0.01 means the blood pressure of the tTOE group *vs.* that of the cTOE group and the concentrated tTOE group); (c) Comparison of the hemolytic toxicity of cTOE and tTOE with the same volume (n = 3; ^**^P<0.01 means the hemolytic percentage of tTOE *vs.* cTOE); (d) Comparsion of the hemolytic toxicity of cTOE and tTOE at the same dose (n = 3; ^**^P<0.01 means the hemolytic percentage of tTOE *vs.* cTOE). (e) SDS-PAGE analysis of cTOE, concentrated tTOE and tTOE. (f) The protein densitometric analysis of the tTOE, concentrated tTOE and cTOE (n = 3).

By one-step alkali denaturation and twice dialysis, the protein concentration of cTOE was reduced from 1.5 to 0.5 mg/ml. After tTOE injection (i.v.) at the dose of 3.3 mg/kg, corresponding to 10 mg/kg of cTOE, the arterial blood pressure decreased from 108 mmHg to 80 mmHg within 10 min in the anesthetized Sprague-Dawley (SD) rats, indicating a retained cardiovascular toxicity, though much lower than that of cTOE. However, when tTOE was injected at the same dose with cTOE (10 mg/kg i.v.), no statistic difference was observed between the hypotensive responses of the two samples (Figure 1a, b). The hemolytic toxicity of tTOE was completely lost (Figure 1c, d). SDS-PAGE displayed multiple bands in cTOE, concentrated tTOE and tTOE (Figure 1e), but the number and density of protein bands were much lower in tTOE than those in cTOE, consistent with the determination of protein concentration (Figure 1e, f). Especially the protein bands were larger than the 92 kDa marker, and the notable protein bands were between 43 kDa and 55 kDa (Figure 1e).

### Effects of tTOE on Cardiac Function Both *in vivo* and *in vitro*

**Figure 2:**
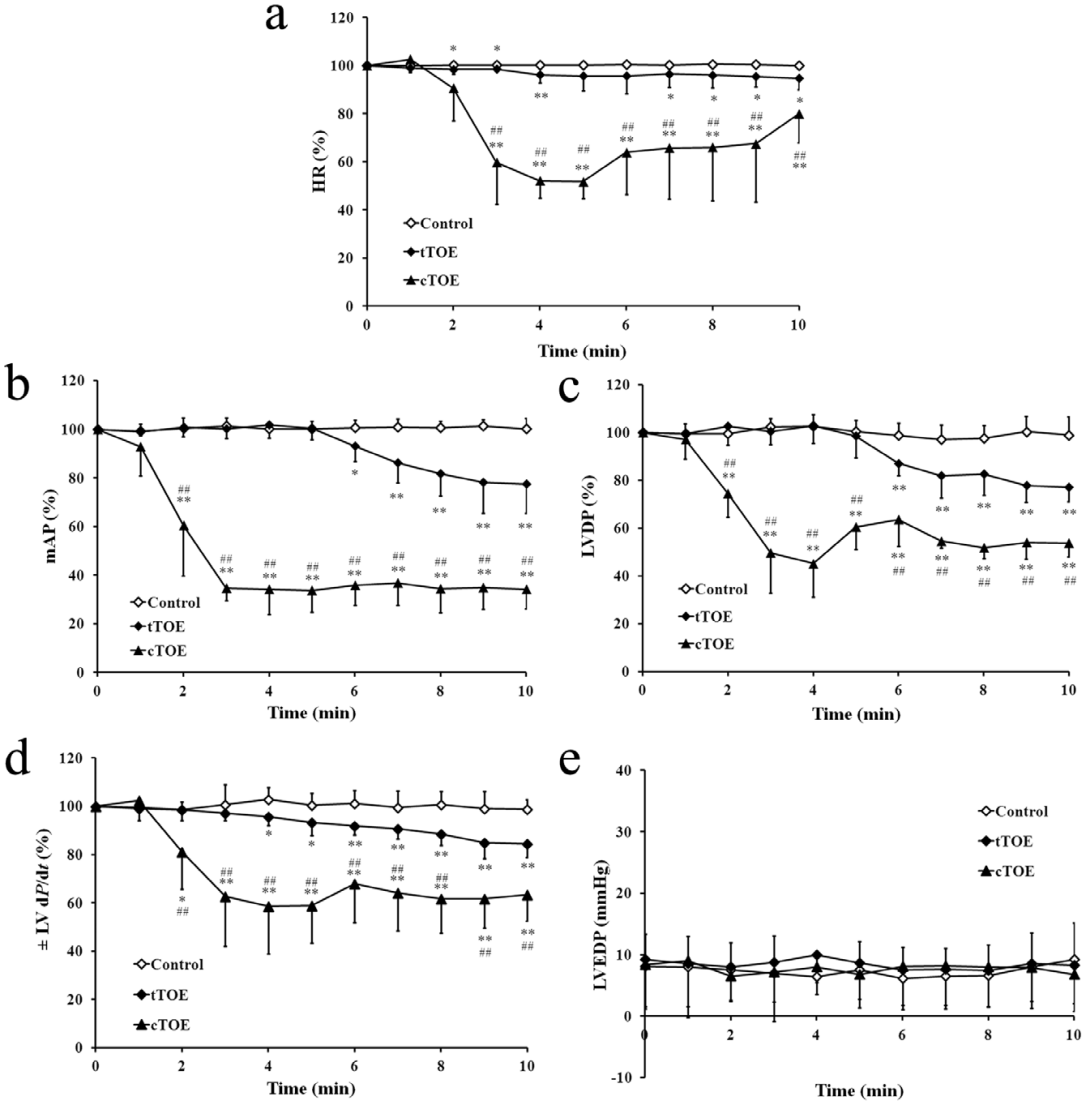
Effects of tTOE (3.3 mg/kg, i.v.) and cTOE (10 mg/kg, i.v.) on cardiac function *in vivo*. (a) HR = heart rate; (b) mAP = mean arterial pressure; (c) LVDP = left ventricular developed pressure; (d) ± LV dP/dt = positive and negative first derivatives of left ventricular pressure; (e) LVEDP = left ventricle end-diastolic pressure. Data are shown as percentage of the pre-injection value (0 min), except that LVEDP is shown as the original value recorded by the biological signal analytic system. Each value is expressed as mean ± SD (n = 8; ^*^P<0.05, ^**^P<0.01 means the cardiac indexes after administration of tTOE or cTOE *vs.* that of solution controls; *^#^P*<0.05, *^##^P*<0.01 means the cardiac indexes after tTOE administration *vs.* that of cTOE).

After tTOE (3.3 mg/kg, i.v.) administration *in vivo*, the indexes of cardiac function, including HR (Figure 2a), mAP (Figure 2b), LVDP (Figure 2c) and ± LV d*P*/d*t* (Figure 2d), declined significantly. However, the effects of tTOE were much weaker than those caused by cTOE (10 mg/kg, i.v.). In addition, the LVEDP did not increase in both tTOE and cTOE groups at any time (Figure 2e).

**Figure 3:**
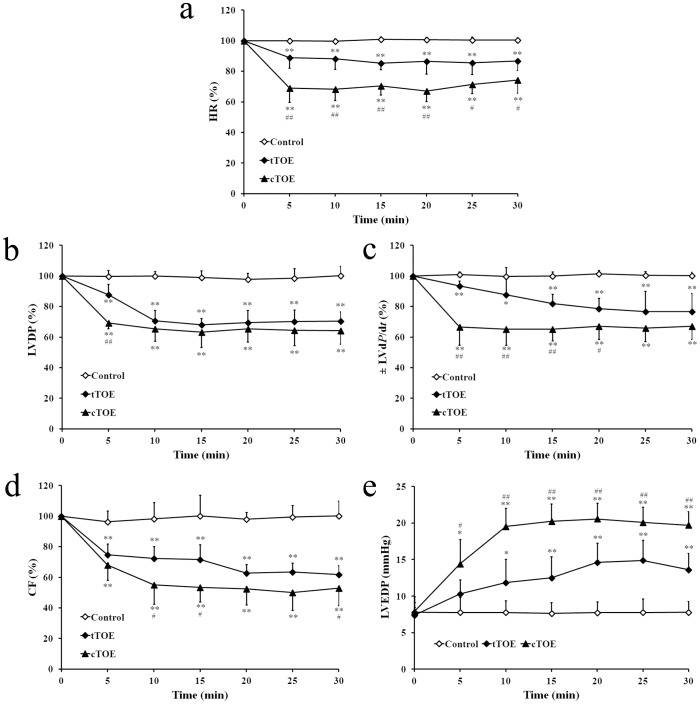
Effects of tTOE (0.3 mg) and cTOE (0.3 mg) on cardiac function *in vitro* using the Langendorff isolated heart model. (a) HR; (b) LVDP; (c) ± LV d*P*/d*t*; (d) CF = coronary flow; (e) LVEDP. Data are shown as percentage of the pre-injection value (0 min), except that LVEDP is shown as the original value recorded by the biological signal analytic system. Each value is expressed as mean ± SD (n = 8; ^*^P<0.05, ^**^P<0.01 means the cardiac indexes after administration of tTOE or cTOE *vs.* that of solutin controls; *^#^P*<0.05, *^##^P*<0.01 means the cardiac indexes after tTOE administration *vs.* that of cTOE).

In the Langendorff isolated heart model, the cardiac indexes, including HR (Figure 3a), LVDP (Figure 3b), ± LV d*P*/d*t* (Figure 3c), and CF (Figure 3d), declined significantly after both tTOE (0.3 mg) and cTOE (0.3 mg) administration, though the discrepancy between tTOE and cTOE groups seemed to be less obvious than that *in vivo*. The LVEDP increased significantly in both groups, with a smaller increase in tTOE group (Figure 3e).

### Effects of tTOE on Arterial Blood Gas Indexes

Both cTOE (10 mg/kg i.v.) and tTOE (3.3 mg/kg i.v.) produced significant effects on arterial blood gas indexes in anesthetized SD rats. The value of pH dropped in both groups without significant statistic difference all the time. SO_2_c (%) did not obviously change within 60 min. PCO_2_ was higher in tTOE group than that in cTOE group while PO_2_ was a little lower in tTOE group. Other blood gas indexes, including HCO_3_^−^, HCO_3_std, TCO_2_, BEecf and BE (B), all decreased significantly within 60 min after administration of tTOE, although their respective decreases were lower than those by cTOE (Table 1).

**Table 1:** Effects of cTOE and tTOE on arterial blood gas indexes in anesthetized SD rats.

		Direct indexes			Indirect indexes					
		pH	PCO_2_(mmHg)	PO_2_(mmHg)	HCO_3_^−^(mmol/L)	HCO_3_std(mmol/L)	TCO_2_(mmol/L)	Beecf(mmol/L)	BE(B)(mmol/L)	SO_2_c(%)
0 min	cTOE	7.38±0.02	48±5	76±9	28.7±2.4	27.0±1.6	30.2±2.6	3.6±2.5	2.7±2	95±2
	tTOE	7.37±0.03	48±4	78±10	27.5±3.1	25.9±2.4	28.9±3.2	2.1±3.5	1.4±3	95±2
5 min	cTOE	7.30±0.06	43±3	84±8	21.7±2.6^**^	21.7±2.1^**^	23.1±2.6^**^	−6.3±3.3^**^	−6±3.4^**^	95±2
	tTOE	7.31±0.04^**^	50±3^##^	84±14	25.0±1.5^#^	23.7±1.6	26.5±1.4^#^	−0.9±2.1^##^	−1.6±2.0^#^	95±2
10 min	cTOE	7.26±0.03^**^	42±4^*^	92±3^**^	17.1±2.5^**^	17.1±2.0^**^	18.4±2.6^**^	−10.4±3.0^**^	−10.0±2.6^**^	96±1
	tTOE	7.25±0.03^**^	55±10^#^	82±18	24.9±3.9^##^	22.3±2.33^*##^	26.4±4.2^##^	−2.1±3.4^*##^	−2.9±2.7^*##^	94±3
20 min	cTOE	7.26±0.05^**^	44±2^*^	89±7^**^	19.0±1.8^**^	18.8±2.0^**^	20.3±1.9^**^	−8.1±2.1^**^	−7.8±1.9^**^	96±1
	tTOE	7.28±0.01^**^	52±7^#^	86±9^*^	24.4±3.6^##^	22.7±2.1^*##^	26.0±3.0^##^	−2.1±3.1^*##^	−2.8±2.7^*##^	95±1
60 min	cTOE	7.28±0.02^**^	36±3^*^	85±7^*^	15.9±2.1^**^	17.0±2.3^**^	17.0±2.2^**^	−11.1±3.0^**^	−10.2±3.0^**^	95±2
	tTOE	7.30±0.05^**^	46±11	78±13	21.9±3.1^**##^	21.1±1.8^**##^	23.2±3.4^**##^	−4.7±3.0^**##^	−4.8±2.4^**##^	95±1

Data are presented as mean ± SD (n = 6, ^*^P<0.05, ^**^P<0.01 *vs.* 0 min; *^#^P<*0.05, *^##^P<*0.01 tTOE *vs.* cTOE).

After tTOE (3.3 mg/kg i.v.) administration, Na^+^ rapidly decreased while K^+^ rapidly increased to the maximum at 5 min (7.9±1.6 mmol/L), then partially recovered respectively, which was similar to the changes caused by cTOE (10 mg/kg i.v.) except that the K^+^ change was much smaller in tTOE group. Ca^2+^ did not change all the time by tTOE, but declined notably by cTOE. Lac, which increased significantly by cTOE, did not change notably by tTOE. Glu increased quickly and retained a relatively high level within 60 min by both tTOE and cTOE. Other indexes, including Hct% and THbc, did not change significantly (Table 2).

**Table 2:** Effects of cTOE and tTOE on blood indexes as measured by an arterial blood gas analyzer in anesthetized SD rats.

		Na^+^(mmol/L)	K^+^(mmol/L)	Ca^2+^(mmol/L)	Ca^2+^(7.4)(mmol/L)	Glu(mmol/L)	Lac(mmol/L)	Hct(%)	THbc(g/dL)
0 min	cTOE	143±1	3.7±0.2	1.19±0.03	1.18±0.03	8.3±1.0	2.5±0.4	45±4	14.4±1.0
	tTOE	143±2	3.6±0.2	1.18±0.04	1.17±0.05	8.1±1.3	2.7±0.7	47±4	14.8±1.1
5 min	cTOE	130±4^**^	12.2±1.7^**^	0.79±0.10^**^	0.75±0.09^**^	9.0±2.8	5.2±1.4^**^	51±5	15.8±1.5
	tTOE	134±3^**#^	7.9±1.6^**##^	1.23±0.09^##^	1.18±0.08^##^	11.1±3.9^*^	3.6±1.4^#^	42±3^*##^	12.8±1.0^*##^
10 min	cTOE	134±3^**^	10.2±1.7^**^	0.82±0.14^**^	0.77±0.14^**^	11.3±3.8^*^	6.1±0.6^**^	52±3^*^	16.7±0.7^**^
	tTOE	133±3^**^	7.1±1.1^**##^	1.18±0.07^##^	1.11±0.05^*##^	9.7±0.9^*^	3.7±1.4^##^	44±5^#^	13.6±1.8^##^
20 min	cTOE	134±4^**^	6.5±0.8^**^	0.99±0.16^*^	0.99±0.09^**^	10.5±2.6^*^	4.8±0.8^**^	51±4	15.7±1.4
	tTOE	134±2^**^	6.9±0.6^**^	1.24±0.10^#^	1.18±0.08^##^	11.2±1.4^*^	3.6±0.9^#^	48±2	14.9±0.6
60 min	cTOE	134±5^**^	5.0±0.8^**^	1.21±0.06	1.15±0.07	10.4±1.3^*^	5±0.8^**^	48±2	15.1±0.8
	tTOE	136±4^**^	5.3±0.9^**^	1.20±0.11	1.15±0.09	9.0±1.3	2.5±1.0^##^	45±4	14.1±1.3

Data are presented as mean ± SD (n = 6, ^*^P<0.05, ^**^P<0.01 *vs*. 0 min; *^#^P<*0.05, *^##^P<*0.01 tTOE *vs.* cTOE).

### Histopathological Analysis

**Figure 4:**
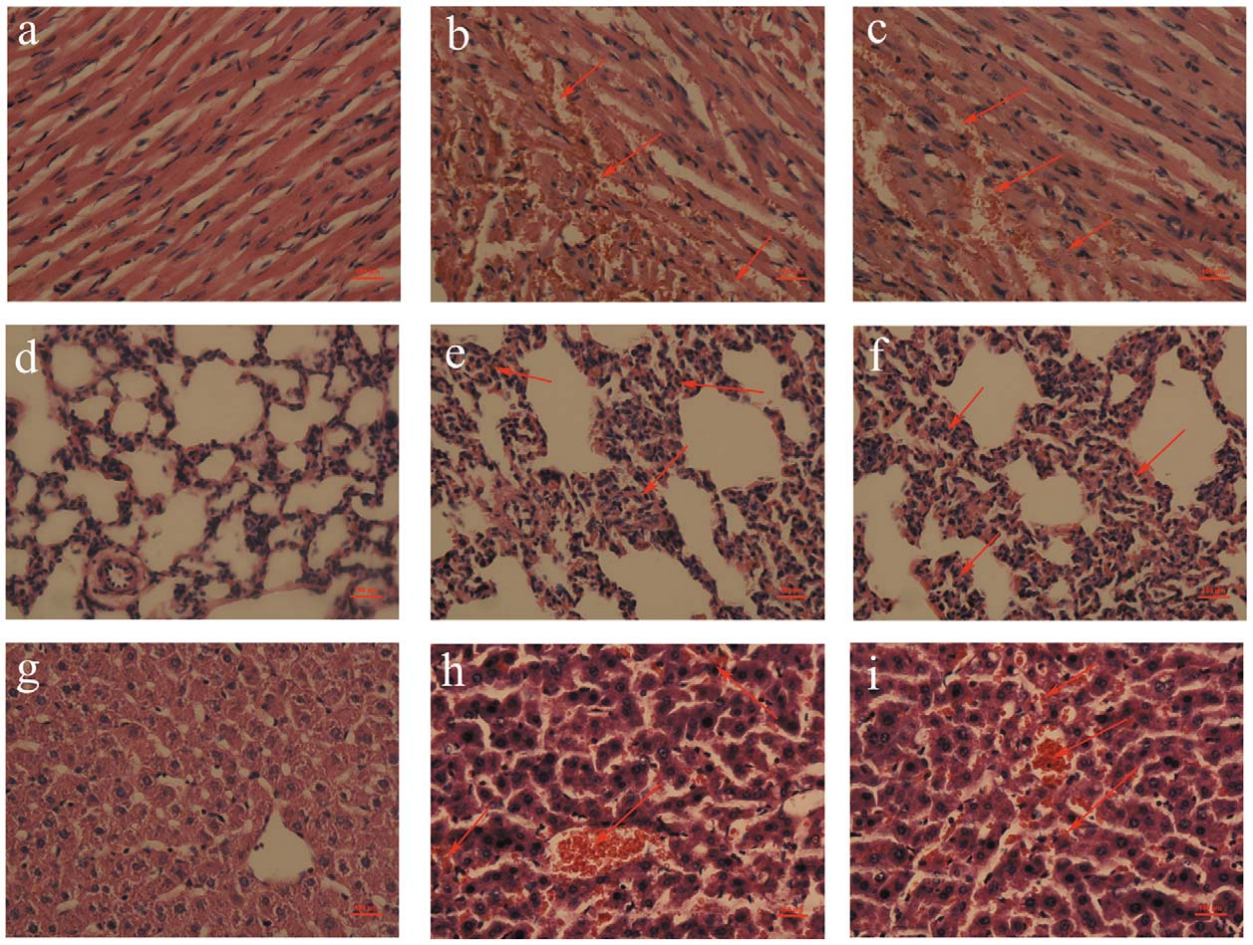
Pictures are HE-stained organs of SD rats, including heart, lung and liver after cTOE (10 mg/kg i.v.) or tTOE (3.3 mg/kg i.v.) administration for 60 min, magnified by 400 × (n = 6). (a) Normal heart tissues; (b) Hemorrhage and denaturation are present in the heart by cTOE; (c) Hemorrhage and denaturation are present in the heart by tTOE; (d) Normal pulmonary tissues; (e) Acute congestion is present in the lung by cTOE; (f) Acute congestion is present in the lung by tTOE; (g) Normal liver tissues; (h) Acute congestion is present in the liver by cTOE; (i) Acute congestion is present in the liver by tTOE (Arrows indicate the lesions).

After administration of cTOE (10 mg/kg i.v.) or tTOE (3.3 mg/kg i.v.), a variety of tissue lesions emerged. Hemorrhage and denaturation were observed in the myocardium (Figure 4a, b, c), and acute congestion was detected in both lung (Figure 4d, e, f) and liver (Figure 4g, h, i) tissues for both the samples.

### Transmission Electron Microscopy

**Figure 5:**
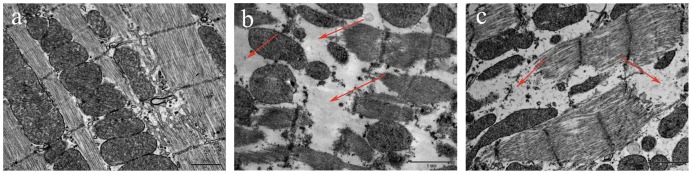
Ultrastructure of rat cardiomyocytes after tTOE (3.3 mg/kg i.v.) or cTOE (10 mg/kg i.v.) administration for 60 min (n = 3). (a) Normal rat cardiomyocyte, sarcomeric structures with Z-lines, mitochondria with crista are integrated and clear in control cardiomyocytes; (b) Sarcomeric filaments are disrupted, dissolved and disappeared after cTOE treatment;.(c) Sarcomeric filaments are disrupted, dissolved and disappeared after tTOE treatment (Arrows indicate the lesions) (scale bar = 1 µm).

Transmission electron microscopy revealed obvious changes in cardiomyocytes 60 min after administration of cTOE (10 mg/kg i.v.) or tTOE (3.3 mg/kg, i.v.). Compared to the normal control (Figure 5a), most sarcomeric structures with Z-line were still integrated accompanied with the disruption, dissolution and disappearance of parts of the sarcomeric filaments in both samples (Figure 5b, c).

### Ventricular Myocytes

**Figure 6:**
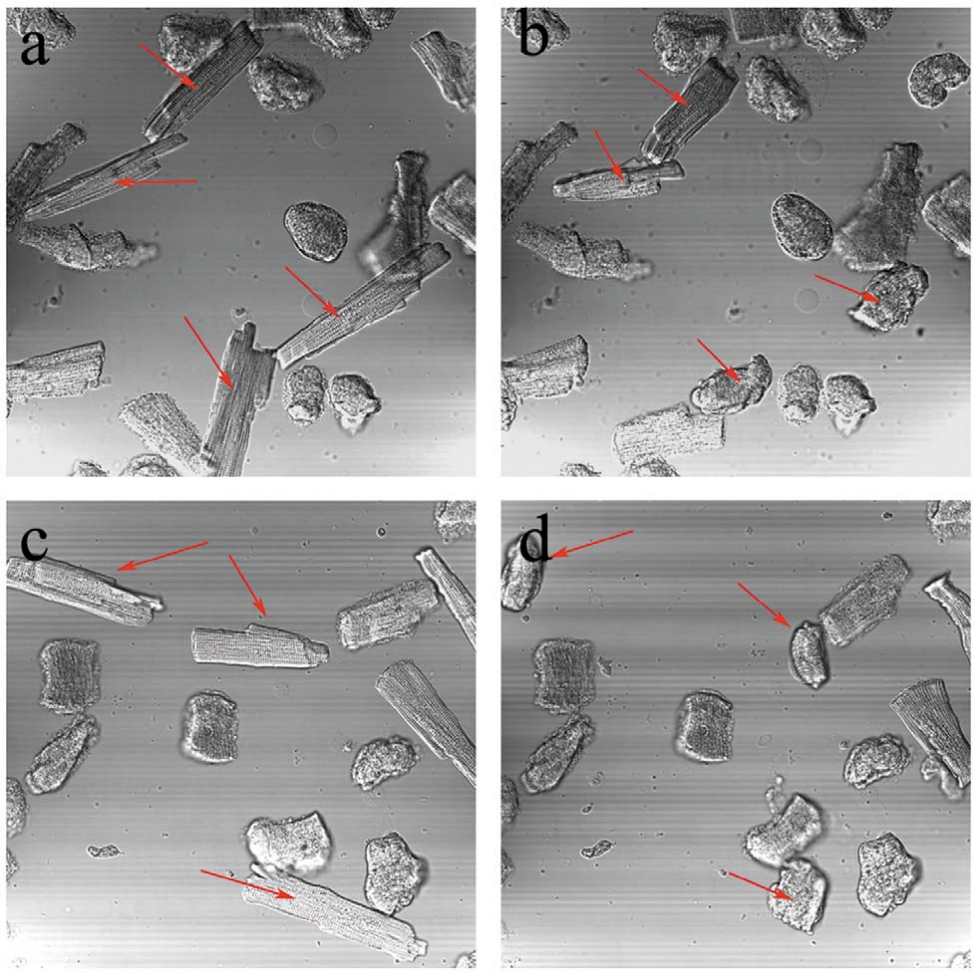
Effects of cTOE (100 µg/ml) and tTOE (30 µg/ml) on isolated rat ventricular myocytes under prescribed vision with a laser scanning confocal microscope (n = 3, magnified by 400 ×). (a) Normal control before cTOE administration; (b) 30 min after cTOE treatment; (c) Normal control before tTOE administration; (d) 30 min after tTOE treatment (Arrows indicate the dead cells).

After treatment with cTOE (100 µg/ml) or tTOE (30 µg/ml), the ventricular myocytes showed a series of changes with a gradual emergence of deformation and ultimate death within 30 min for both samples (Figure 6a, b, c, d).

## Discussion

Researchers have been working on the purification of the cardiovascular toxic component and the mechanism of cardiovascular toxicity for more than 60 years [1], [3]. Unfortunately, no substantial advances have been made, and the purification of the cardiovascular toxic component has become the bottleneck in the field of research on jellyfish venoms [1], [3]. Our laboratory has been carrying out the purification of the cardiovascular toxic component of jellyfish venoms and exploring the mechanism of cardiovascular toxicity for several years [9], [34], and we become aware that the lack of pre-treatment of the venom sample is one of the important causes for the small advances.

We have previously investigated the physiochemical factors influencing both the cardiovascular and hemolytic toxicities of cTOE, and found an obvious tolerant discrepancy to the alkaline environment between the two toxicities, where the cardiovascular toxicity retained well between pH 7 and 11 [32] while the hemolytic toxicity showed significant decrease at pH 8 or more [33]. Moreover, we also found that plenty of precipitates were produced under two conditions: one was alkaline treatment within pH 11, and we chose pH 10.5 with NaOH (0.01 mol/L); the other was the different dialysis solutions standing in a relative long time, and we chose the Tris-HAc 9.5 and HAc 6.0 as the dialysis solutions. So, the tTOE without the hemolytic toxicity was obtained by an alkaline denaturation followed by dialysis against two different buffer solutions in the present study.

After treatment, the protein concentration of tTOE was about 1/3 of cTOE. The hemolytic toxicity of tTOE was lost while its cardiovascular toxicity was well retained. In the SDS-PAGE image, dozens of protein components were observed in tTOE and cTOE respectively, though the bands in tTOE were fewer than those in cTOE. Because the missing components, as well as the cTOE and tTOE, all contained dozens of proteins, it was inadequate to define the hemolytic toxic component for the moment. However, a notable difference in the protein bands between 40 to 50 kDa was observed in the SDS-PAGE image, and we speculated that the notably changing bands among this area might be the possible hemolysins of the jellyfish *C. capillata* since the molecules of hemolysins were reported to be between 40 to 50 kDa in a few articles [21], [22], [24]. Other bands with discrepancies, larger than the 92 kDa marker, might be recognized as the jellyfish tissue proteins since the toxic components are likely to be relatively small secretory protein molecules. As many protein bands were still located in tTOE, the cardiovascular toxic component could not be speculated as well. But it can be a significant step forward to successfully carry out such a pre-treatment in this stage to facilitate the final purification followed by different chromatography such as ion-exchange, size-exclusion and so on.

The characterization of the cardiovascular toxicity of tTOE was further investigated. The indexes of cardiac function, including HR, mAP, LVDP and ± LV dP/dt, declined within 10 min both *in vivo* and *in vitro*, indicating a heart failure appeared. The disparity of LVEDP can be possibly explained by the fact that the solution in the Langendorff isolated heart model was retrogradely perfused through the coronary artery and the left ventricle was mainly influenced. So LVEDP increased notably *in vitro*. When the jellyfish venom was injected *in vivo*, both the left and right ventricles were damaged. A whole heart failure appeared, and LVEDP was not changed all the time. Defects by histopathological and electron microscopic examinations in the heart are consistent with heart failure, but other defects appeared in lung and liver are difficult to be discerned whether they are a consequence of heart failure or the direct effect of tTOE itself. Death of the isolated ventricular myocytes contributed to the demonstration of a direct heart injury by tTOE.

However, the heart damage by tTOE was slighter than that by cTOE, though the concentrated tTOE with the equal concentrations of cTOE and the same hypotensive responses. We suppose that these discrepancies were related to the inactivation of hemolytic activity, which could increase the sensitivity of the heart to the cardiovascular toxic component by elevation of the K^+^ and acidosis [10], [31] or direct cytotoxicity. Another possible reason for the drop in potency of the cardiovascular toxicity of tTOE might be the partial loss or dilution of the cardiovascular toxic component in the process of alkaline denaturation and dialysis.

Results of blood gas monitoring indicated that both tTOE and cTOE caused an extensive and serious imbalance in the blood gas indexes and electrolytes *in vivo*. When tTOE was administered, the hemolytic indexes remained normal or decreased significantly, further supporting the conclusion that the hemolytic toxicity was inactivated by the alkaline denaturation and dialysis. An interesting phenomenon is that the cations seem to transport through the cell membrane freely after cTOE treatment. The blood Na^+^ and Ca^2+^ decreased while the blood K^+^ increased, but they remained normal or only changed mildly after the hemolytic toxicity was inactivated, implying that hemolysin in cTOE may act as a non-selective cation pore-former on cell membrane, consistent with the previous sequence analysis [27], [28], [29], [30]. In addition, although the K^+^ concentration and acidosis were much lower and later than those in cTOE group, the increased K^+^ and acidosis still appeared in tTOE group. From the order of the changes happening, we can see that the K^+^ increase was a bit earlier than the acidosis, implying that the acidosis might be caused by K^+^ elevation through the H^+^-K^+^ exchange mechanism. So the acidosis maybe increased secondarily to the K^+^ elevation.

In conclusion, by the treatment of one-step alkaline denaturation and twice dialysis, the hemolytic toxicity was inactivated completely from the cTOE with its cardiovascular toxicity well retained. Thus, the cardiovascular toxicity of cTOE can exist independently of hemolytic toxicity and the tTOE can be employed as the better venom sample for further purification and mechanism research on the jellyfish cardiovascular toxic proteins.

## Materials and Methods

### Preparation of cTOE and tTOE from the Jellyfish *C. capillata*

Specimens of *C. capillata* were collected in July 2009 on the Sanmenwan coast of the East China Sea in Zhejiang Province, China. Jellyfish catching was permitted by the East China Sea Branch, State Oceanic Administration, People’s Republic of China. The removed tentacles were preserved in plastic bags on dry ice and immediately shipped to Shanghai, where the samples were frozen at −70°C until use. The cTOE was prepared following the method described by us and other authors [32], [35]. Briefly, frozen tentacles were thawed at 4°C and immersed in filtered seawater at the mass: volume ratio of 1∶1 to allow autolysis of the tissues for 4 days. The mixture was stirred for 30 min twice daily. The autolyzed mixture was centrifuged at 10 000 × *g* for 15 min thrice. The resultant supernatant was the cTOE.

The cTOE was set to pH 10.5 by NaOH (0.01 mol/L), allowed to stand for 4 h, centrifuged at 10 000 × *g* for 15 min, and the sediments were removed. The resultant supernatant was dialyzed against 0.02 mol/L acetic acid (HAc, pH6.0) for 24 h, replaced by 0.02 mol/L Tris-HAc (pH 9.5) for another 24 h, and then centrifuged at 10 000 × *g* for 15 min to remove the sediments. The resultant supernatant was the tTOE.

All the procedures were performed at 4°C or in an ice bath. Before being used for injection, the cTOE and tTOE were centrifuged at 10 000 × *g* for 15 min to remove the sediments, followed by dialysis against PBS (0.01 mol/L, pH 7.4) for 8 h. The protein concentration in the preparations was determined with the Bradford method [36], with fetal bovine serum as the standard.

### Determination of Cardiovascular and Hemolytic Toxicities

Male SD rats (200±20 g, provided by the Laboratory Animal Center of the Second Military Medical University, Shanghai) were anesthetized by 25% urethane (1.0 g/kg, i.p.). An arterial cannula, connected to a pressure sensor, was inserted into the left femoral artery to monitor and record the changes of blood pressure by an MPA-2000 bio-signal analysis system (Alcott Biotech, Shanghai), and the cTOE or tTOE was administered through a catheter placed in the right external jugular vein.

A heparinized blood sample was drawn by a syringe through a catheter that was inserted into the left femoral artery of the anesthetized SD rats. The blood was diluted in PBS, centrifuged (800 × *g*) for 10 min, and the supernatant was removed. This washing procedure was repeated three times, and finally the sediments were re-suspended in the same buffer to an erythrocyte concentration of 2%. The erythrocyte suspension was incubated with cTOE or tTOE at 37°C for 30 min, centrifuged at 800 × *g* for 10 min to sediment both intact erythrocytes and ghosts. An aliquot of the supernatant was then taken and the optical density (OD) was spectrophotometrically measured at 414 nm to determine the percentage of hemoglobin released from the lysed erythrocytes. The hemolytic activity of cTOE or tTOE was expressed as % absorbance compared with that observed after maximal lysis under saponin (25 µg/ml). The supernatant of untreated erythrocyte suspension was taken as the control and subtracted. All the animal experiments were approved by the Ethics Committee of the Second Military Medical University.

### SDS-PAGE

In the samples of cTOE and tTOE, 4 × loading buffer was added for subsequent electrophoresis. The sample-mixtures were heated at 100°C for 10 min and then cooled in ice-cold water for 15 min. The running gel (1.5 mm) of 12% polyacrylamide and a broad range molecular weight marker (17–170 kDa) were selected. The running voltage was 80 V and 150 V for the stacking and running gels, respectively. After electrophoresis, the gels were washed three times in distilled water, dyed with Coomassie R-250 staining (0.1% Coomassie R-250, 50% ethanol, and 10% acetic acid) for 20 min, and the gels were finally de-stained in 250 mmol/L KCl for several hours until the protein bands were clearly visualized. The densitometric analysis of the image of SDS-PAGE was performed by GeneSnap 6.08.

### Determination of Cardiac Function *in vivo*

Tracheotomy was performed on the anesthetized male SD rats (200±20 g) to make spontaneous ventilation easier. A heparinized catheter was inserted into the external jugular vein for intravenous administration of cTOE or tTOE. Mean arterial pressure (mAP) was measured through a catheter that was inserted into the left femoral artery. The catheter was connected to a pressure transducer. Left ventricular pressure (LVP) was measured through a catheter that was introduced through the right carotid artery into the left ventricular cavity. The catheter was connected to another transducer. The first derivative of LVP (± LV d*P*/d*t*), an index of cardiac systolic and diastolic function, was derived from differentiating the signal of LVP using an electronic differentiator. Left ventricular developed pressure (LVDP) was calculated from the following equation: LVDP (mmHg) = LVP_max_ – LVP_min_. An MPA-2000 bio-signal analysis system was used to monitor and record the changes of HR, mAP, LVDP, LVEDP and ± LV d*P*/d*t*.

### Determination of Cardiac Function *in vitro*

Male SD rats (300±20 g) were anesthetized with a mixture of urethane (1.0 g/kg, i.p.) and heparin (400 IU/kg, i.p.). The heart was rapidly excised and placed into ice-cold KH solution. Then the heart was mounted onto a Langendorff apparatus and retrogradely perfused using a peristaltic pump (MPA-2000, Alcott Biotech, Shanghai) with warm KH solution (37±0.5°C, pH 7.35–7.40). The KH solution, equilibrated with a gas mixture (95% O_2_ and 5% CO_2_), was adjusted at a constant flow of 12–16 ml/min, thus maintaining an initial perfusion pressure of 60–80 mmHg. A ﬂuid-filled balloon was introduced into the left ventricle through a polyethylene cannula. The initial balloon volume was set to generate the LVEDP of 2–8 mmHg. After at least 20 min of stabilization, cTOE or tTOE was injected in bolus, and the cardiac indexes, including HR, LVDP, LVEDP, and ± LV d*P*/d*t*, were recorded by the MPA-2000 bio-signal analysis system. The coronary flow (CF) was measured by weighing the effluent from the Langendorff circuit.

### Hematological Indexes Determined by an Arterial Blood Gas Analyzer

Tracheotomy was performed on male SD rats (220±20 g) to make spontaneous ventilation easier. A heparinized catheter was inserted into the external jugular vein for intravenous administration of cTOE or tTOE. An arterial blood sample was drawn through a catheter that was inserted into the left femoral artery by a heparinized syringe. Afterwards, the syringe was sealed quickly with a rubber stopper. Hematological indexes, including blood gases (Direct indexes: pH, PCO_2_ and PO_2_; Indirect indexes: HCO_3_^−^, HCO_3_std, TCO_2_, BEecf, BE (B) and SO_2_c (%)), electrolytes (Na^+^, K^+^ and Ca^2+^), hematocrit (Hct), glucose (Glu) and lactic acid (Lac), were measured within 30 min by an arterial blood gas analyzer (GEM Premier 3 000, International Laboratory, USA). Measurements were divided into six groups: 0 (pre-administration), 5, 10, 20, 60 min after administration of cTOE or tTOE.

### Histopathological Analysis

The organs (heart, lung and liver) were collected from the SD rats after treatment with cTOE or tTOE for 60 min, and then fixed in 10% formalin. The tissues were embedded in paraffin, sectioned into pieces of 6 µm thicknesses and stained with hematoxylin and eosin (HE) for microscopy examination.

### Transmission Electron Microscopy

The hearts were collected from the SD rats after treatment with cTOE or tTOE for 60 min, excised into tablets less than 1 mm^3^ and fixed in 4% paraformaldehyde (PFA), rinsed twice in 0.1 mol/L sodium phosphate, followed by 3% glutaraldehyde for 6 h. Then the specimens were fixed by 1% osmium tetroxide for 20 h, rinsed twice and followed by dehydration and embedment. The sections (100–120 nm) were cut and stained with lead citrate and uranyl acetate. Microscopic examination was performed with a transmission electron microscope (Hitachi H-800) at the Electron Microscope Laboratory of the Second Military Medical University, Shanghai, China.

### Ventricular Myocytes

Ventricular myocytes were isolated from SD rat according to the previously described technique with minor modifications [37]. Initially, the heart was perfused with a normal Tyrode solution containing 1.8 mmol/L Ca^2+^. After stabilization, the heart was then perfused for 4 min with a cell isolation solution (mmol/L: NaCl 130, KCl 5.4, MgCl_2_ 1.4, NaH_2_PO_4_ 0.4, HEPES 5, glucose 10, taurine 20 and creatine 10, set to pH 7.3 with NaOH) containing 0.1 mmol/L EGTA, and then for 6 min with a cell isolation solution containing 0.05 mmol/L Ca^2+^, 0.75 mg/ml collagenase II and 0.075 mg/ml protease XIV. After this time, the ventricle was excised from the heart, minced, and gently shaken in collagenase-containing isolation solution supplemented with 1% BSA. The cells were filtered from this solution at 4-min intervals and re-suspended in 0.75 mmol/L Ca^2+^-containing isolation solutions. Isolated rat ventricular myocytes were treated by cTOE or tTOE for 30 min, examined with a laser scanning confocal microscope (LeicaCTS SP2).

### Data Analysis

One-way analysis of variance (ANOVA) was used. In all cases, statistical significance was indicated by *P*<0.05. All quantitative data are expressed as mean ± SD.
